# 536. Development of post acute sequelae of SARS-Cov-2 infection (long Covid) in outpatient receiving monoclonal antibodies

**DOI:** 10.1093/ofid/ofad500.605

**Published:** 2023-11-27

**Authors:** Amelia Neto, Vivek Kak

**Affiliations:** PIIC Center, Missisauga, Ontario, Canada; PIIC Center, Missisauga, Ontario, Canada

## Abstract

**Background:**

The development of post-acute sequelae of SARS-Cov-2 infection (long COVID), is a complex condition defined as a persistence of symptoms at least 3 months after initial infection. Although the etiology of long COVID is poorly understood, studies suggest it is related to immune dysregulation following infection. The development of long COVID is unclear with studies suggesting it can occur in up to 15% of individuals, however most of this data focuses on hospitalized patients. We examined the occurrence of long COVID in patients who received the monoclonal antibody (mAb) treatment at an outpatient infusion center.

**Methods:**

We performed a study of high-risk symptomatic COVID patients who received the mAb treatment at an OP center from April 2021-22. Patients who received the mAb treatment were characterized based on the following criteria: age > 65, BMI > 25, or comorbidity. A follow up closed-ended survey was used to determine if patients experienced any of the following long COVID symptoms: fatigue, exertion intolerance, persistent cough or chest pain, dyspnea, brain fog, headaches, issues sleeping, myalgia, or loss of smell/taste.

**Results:**

We called 1100 patients, yielding 615 responses, of which 57.7% (n = 355) fully recovered, while 42.3% (n = 260) reported experiencing long COVID. From the subset of individuals who experienced long COVID, 4.6% (n = 12) were hospitalized. In addition, 50% (n = 129) of long COVID patients reported having 3 or more symptoms, with fatigue, brain fog, and loss of smell/taste being the most prevalent. The characteristics and outcomes of patients who received the mAb treatment are shown in Table 1.

**Figure:**
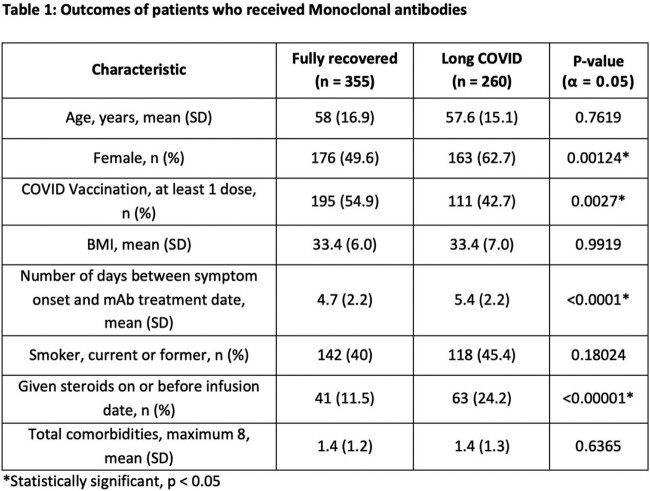

**Conclusion:**

Long COVID was documented in almost half of high-risk symptomatic patients suggesting a higher incidence compared to the literature, despite a low hospitalization rate. Female sex, unvaccinated patients and those who received the mAb later were more likely to develop long COVID. Patients given steroids on/before the date of their mAb also had more long COVID. Predictors of long COVID based on our data includes female sex, vaccination status, and the administration/timing of immune modulators. Modulation of the immune system is likely important in controlling the development of long COVID. These results need to be validated in larger cohorts.

**Disclosures:**

**All Authors**: No reported disclosures

